# Association between air temperature and risk of hospitalization for genitourinary disorders: An environmental epidemiological study in Lanzhou, China

**DOI:** 10.1371/journal.pone.0292530

**Published:** 2023-10-11

**Authors:** Runping Zhang, Wancheng Zhang, Jianglong Ling, Jiyuan Dong, Li Zhang, Ye Ruan

**Affiliations:** School of Public Health, Lanzhou University, Lanzhou, People’s Republic of China; Kanazawa University: Kanazawa Daigaku, JAPAN

## Abstract

**Objectives:**

The aim of this study was to investigate the relationship between air temperature and the risk of hospitalization for genitourinary disorders.

**Methods:**

Distributed lag non-linear models (DLNM) were used to estimate the association between air temperature and the risk of hospitalization for genitourinary disorders, with subgroup analysis by gender and age to identify the susceptible population of temperature-sensitive genitourinary system diseases.

**Results:**

Low mean temperature (MT) (RR = 2.001, 95% CI: 1.856~2.159), high MT (RR = 2.884, 95% CI: 2.621~3.173) and low diurnal temperature range (DTR) (RR = 1.619, 95% CI: 1.508~1.737) were all associated with the increased risk of hospitalization for genitourinary disorders in the total population analysis, and the high MT effect was stronger than the low MT effect. Subgroup analysis found that high MT was more strongly correlated in male (RR = 2.998, 95% CI: 2.623~3.427) and those <65 years (RR = 3.003, 95% CI: 2.670~3.344), and low DTR was more strongly correlated in female (RR = 1.669, 95% CI: 1.510~1.846) and those <65 years (RR = 1.643, 95% CI: 1.518~1.780).

**Conclusions:**

The effect of high MT on the risk of hospitalization for genitourinary disorders is more significant than that of low MT. DTR was independently associated with the risk of hospitalization for genitourinary disorders.

## 1. Introduction

The Sixth Assessment Report of the United Nations Intergovernmental Panel on Climate Change points to growing evidence that climate change is already causing many weather and climate extremes in all regions of the world, that extreme weather events such as heat waves are being observed, and that some 3.3–3.6 billion people are living in environments that are highly vulnerable to climate change [1]. Global warming is one of the types of climate change that results from the accumulation of the greenhouse effect, which leads to an imbalance between the energy absorbed and emitted by the geothermal system, and the accumulation of energy in the geothermal system, which leads to an increase in temperature, resulting in global warming. Gradually, attention is being paid to the association between temperature and disease. Studies have now been conducted to prove that temperature is associated with the occurrence of multi-system diseases such as respiratory [2], circulatory [3] and neurological [4].

A study of different regions in Korea found that low temperatures led to an increased risk of acute kidney injury admissions in older male [5], that the overall cumulative RR of acute kidney injury in older male was high and statistically significant for high temperatures [6], and that the relative risk of developing urinary stones increased by 1.017 for every 1°C above the threshold temperature (95% CI: 1.010~1.024) [7]. In a study of Australia, a 1°C increase in temperature during the hot season was found to be associated with a 3.3% increase in hospital admissions for urological disorders (95% CI: 2.9%~3.7%), with a stronger association in male and the elderly [8]. A mate analysis of the literature on kidney disease during 1990–2020 found that high temperature had the greatest impact on the risk of developing urolithiasis, particularly in male and in those <65 years of age [9]. Stronger association of diurnal temperature range (DTR) with emergency admissions for genitourinary disorders in the elderly in a study of the Beijing population [10]. In a study of Taiwanese female, the results showed a positive association between the incidence of acute pyelonephritis and mean monthly temperature [11]. The main mechanisms causing genitourinary disorders in humans at high temperatures are related to a range of responses including dehydration of the genitourinary system, rhabdomyolysis [12], local ischaemia, hypoxaemia and ATP depletion, and increased sodium reabsorption [13]. The genitourinary system is made up of the kidneys, ureter, bladder, urethra, internal and external genitalia and the associated blood vessels and nerves. Genitourinary disorders consist of diseases of the urinary and genital tracts. The number of hospital admissions for genitourinary disorders in China is on a year-on-year rise, having increased from 3.9% in 2008 to 8.2% in 2018 [14]. Although studies have previously been conducted on genitourinary disorders, most of them have simply used ambient temperature, maximum and minimum temperatures [15], DTR has emerged as an important indicator for observing temperature changes and provides more useful information than using daily maximum and daily minimum temperatures to observe the association between temperature and disease, so we conducted the following study using mean temperature (MT) and DTR. MT is the average of multiple temperature data measured over a time period (in this study, one day). MT can be used to describe the overall temperature situation in an area or at a particular time period. DTR is the difference in temperature between daytime and nighttime during the course of a day. It is derived by comparing the difference between the highest daytime temperature and the lowest nighttime temperature. DTR can reflect the magnitude of temperature change in an area over time.

Lanzhou also has unique climatic characteristics compared to other Chinese cities. The spring and summer boundaries are indistinct, with short, high temperatures in summer, rapid cooling in autumn, long, cold winters, stable atmospheric formations, severe inversions and the heat island effect of urbanization. As Lanzhou is deep inland in the northwest, it is not easily accessible by oceanic warm and humid air currents and has few opportunities for rainfall. The climate in most areas is dry, with a very continental temperate monsoon climate, so the relationship between air temperature and genitourinary disorders in Lanzhou needs to be studied.

This study was conducted by collecting inpatient treatment data and meteorological data concerning genitourinary system disorders from all hospitals in four main urban districts of China city of Lanzhou (Chengguan, Qilihe, Xigu and Anning districts) from 2014 to 2020. The effect of MT and DTR on the overall risk of hospitalization for genitourinary system disorders was first analyzed. Subgroup analysis was then performed based on gender and age to identify potential genitourinary diseases sensitive to air temperature.

## 2. Materials and methods

### 2.1 Study area

Lanzhou City of China is located at mid-latitude (102°36′~104°35′E, 35°34′~37°00′N), the geometric center of China’s geography, in the middle of Gansu Province, a belt-shaped basin city narrows from north to south and long from east to west. The city is surrounded by mountains to the north and south, and the Yellow River meanders through the city from east to west for more than 100 miles. Lanzhou is the only provincial capital city where the Yellow River passes through the center of the city, and the city’s unique and beautiful urban landscape is formed by the mountains and the water, which are still and moving. 2022 saw the city’s resident population reach 4,415,300 with a total geographical area of 13,100 square kilometers and an urban area of 1,631.6 square kilometers. The total area of the city is 1100 square kilometers Composed of four main urban districts (Chengguan District, Qilihe District, Xigu District and Anning District) with a population of 3.8 million people.

### 2.2 Hospitalization and meteorological data

The four main urban districts are conveniently located in the center of Lanzhou City and are surrounded by densely populated residential areas. At the same time the urban hospitals have well-established medical departments and strong technical capabilities to provide medical services to all residents of the city. And >80% of local residents are willing to seek treatment at these hospitals [2]. Therefore, the hospitalization data from these hospitals can be seen as representative of the risk of hospitalization for genitourinary disorders in the region.

Daily inpatient data from 1 January 2014 to 31 December 2020 were obtained from all hospitals in the four main urban districts of Lanzhou and then classified according to the World Health Organization International Classification of Diseases (ICD-10), based on codes (N00-N99) for inpatient attendance information, including age, gender and date of admission. This study conducted in accordance with the Declaration of Helsinki, collected hospitalization data without identifiable personal information. The Institutional Review Board (IRB) of the School of Public Health, Lanzhou University, exempted the study from ethics approval and consent, considering the absence of identifiable participant information. The researchers ensured the privacy and confidentiality of the data throughout the study, adhering to the guidelines and regulations stipulated in the Declaration of Helsinki.

The meteorological data for this study were obtained from the Lanzhou Meteorological Bureau and consisted mainly of MT, daily maximum temperature, daily minimum temperature and daily mean relative humidity (RH). DTR was obtained by subtracting the daily maximum temperature from the daily minimum temperature. Meteorological data were collected at the national meteorological monitoring station (Lanzhou Station) with professional meteorological observation instruments that had been certified by China Metrology, and professional meteorological observers were responsible for data quality audit and control to ensure that the observation data were complete and accurate. We used air temperatures from only one national meteorological monitoring station for the reason that using air temperatures from one station is equivalent to using spatial and temporal modelling of space temperature to assess the impact of assessing city-wide temperature on hospitalization risk [16]. Air pollutant data were obtained from the Lanzhou Ecology and Environment Bureau. The average level of the four air pollutant monitoring stations was used to represent the air pollution status of four main urban districts in Lanzhou. PM_10_, SO_2_ and NO_2_ were calculated using 24-hour average concentrations. Neither meteorological data nor pollutant data were missing.

### 2.3 Statistical analysis

Descriptive statistics were calculated by SPSS 26 and included minimum, maximum, mean, standard deviation (SD), 5th, 25th, 50th, 75th and 95th percentiles for air pollutants, meteorological variables and hospital admissions. Spearman’s correlation analysis was also performed on daily meteorological data for Lanzhou (2014–2020) using SPSS 26.

Previous studies have shown that the impact of meteorological factors on human health is often delayed and has a lag effect. In order to fully capture the effect of MT and DTR on the risk of hospitalization for genitourinary disorders, we used a distributed lag non-linear models (DLNM) based on a quasi-Poisson distribution. The DLNM is based on a "cross-base" function that allows simultaneous estimation of the non-linear effects of each delay, and it shows the relationship between MT, DTR and the risk of hospitalization for genitourinary disorders at each meteorological factor value point and lag point [2]. Although studies have shown that RH is associated with genitourinary disorders, this study focused on the effect of air temperature on genitourinary disorders and the effect of RH was not studied in detail, only allowing it to enter the model as a confounding factor. There are six routinely detected air pollutants in China, and considering that the high collinearity between PM_2.5_ and PM_10_ cannot be included in the model at the same time. At the same time, the relevant literatures were selected PM_10_, SO_2_ and NO_2_ into the model, so we chose PM_10_, SO_2_ and NO_2_ [5, 10]. In addition, we assumed a non-linear relationship between the hospitalization for genitourinary disorders and PM_10_, SO_2_ and NO_2_. And in order to control the potential effect of multicollinearity, the Spearman’s correlation coefficients for each meteorological factor and pollutant were examined, and only factors with the Spearman’s correlation coefficient <0.7 were entered into the model (S1 Table). The degrees of freedom in the model are chosen based on the minimum value of the quasi-Akaike information criterion (Q-AIC) [17] (S2 Table). We also present the Q-AIC values for each parameter in S2 Table. In the following model, the degrees of freedom (df) for MT, DTR, RH, PM_10_, SO_2_ and NO_2_ was set to 4, and the df for calendar time was set to 7/year. The model is based on the R software (version 4.2.2) and the "dlnm" package. The model is based on the following equations:

$$Log(E(Y_{t}))=\alpha +\Sigma cb(MT,df=4)+ns(RH,df=4)+ns({PM}_{10},df=4)+ns({SO}_{2},df=4)+ns({NO}_{2},df=4)+ns(Time,df=7/year)+factor(Dow)+factor(Holiday)$$

$$Log(E(Y_{t}))=\alpha +\Sigma cb(DTR,MT,df=4)+ns(RH,df=4)+ns({PM}_{10},df=4)+ns({SO}_{2},df=4)+ns({NO}_{2},df=4)+ns(Time,df=7/year)+factor(Dow)+factor(Holiday)$$

*Y*_*t*_ is the number of genitourinary disorder admissions on day *t*; *α* denotes the intercept; *t* denotes the day of visit; and *∑cb()* is a two-dimensional cross basis function of lag days and MT and DTR. As suggested by previous studies [18], in the cross-basis function, the lag days are lag 0–21 days (knots are placed on the value of equal spacing), and the lag dimension is a natural cubic spline with 4 df. The knots of the lag dimension are placed equidistantly on the logarithmic scale. And *ns()* denotes the natural cubic spline, with 4 df being used to adjust for RH, PM_10_, SO_2_ and NO_2_; *ns(Time*, *df = 7)* is 7 df per year to control for long-term trends and seasonality; *Dow* is a categorical variable representing the day of the week effect; *Holiday* is a categorical variable representing the holiday effect. To identify potentially susceptible populations, further subgroup analyses were conducted according to gender (male and female) and age (<65 and ≥65 years). In this study we have not explored air temperature at a deeper level of analysis, but have only classified low and high levels. Referring to related studies [6], the minimum mortality temperature (MMT) -12.3°C for MT and 17.9°C for DTR were determined based on the exposure-response relationship between MT, DTR and the risk of hospitalization for genitourinary disorders. And the relative risk (RR) of high (95th percentile level) and low (5th percentile level) levels relative to the MMT were calculated at the time of the cumulative lag effect, and the effect of the factor on the risk of hospitalization for genitourinary disorder was evaluated by the magnitude of the RR value and the range of its 95% confidence interval (CI).

In addition, we also used the following models to study the relationship between air pollutants and the risk of hospitalization for genitourinary diseases:

$$Log(E(Y_{t}))=\alpha +\Sigma cb(X_{t,l})+ns(MT,df=3)+ns(RH,df=3)+ns(Time,df=7/year)+factor(Dow)+factor(Holiday)$$

*Y*_*t*_ is the number of genitourinary disorder admissions on day *t*; *t* denotes the day of visit; *α* denotes the intercept; *∑cb* (*X*_*t*,*l*_)is the air pollutant (PM_10_, SO_2_, and NO_2_) cross-base matrix, *l* is the number of days lagged. The relationship for the air pollutant-genitourinary disease dimension was fitted with a natural cubic spline function with df of 3. The lag dimension was also fitted with a natural cubic spline function with df of 3. And *ns()* denotes the natural cubic spline, with 3 df being used to adjust for *MT* and *Rh* with previous studies [19]; *MT* is the mean temperature on day *t*; *Rh* is the relative humidity on day *t*. In the current study, we chose 7 lag days as our model. We examined the effects of single day lags (from lag0 to lag7) and cumulative lags (lag0-1 to lag0-7) of air pollutants (PM_10_, SO_2_, and NO_2_) on total genitourinary disorder admissions. The results of the analysis were expressed as RR and its 95% CI in association with 10 μg/m^3^ increase in each pollutant.

### 2.4 Sensitivity analysis

To assess the stability of the model, sensitivity analyses were conducted by varying the degrees of freedom of the time variables (df = 6, 7, 8, 9), the maximum lag days (max lag = 21, 14, 28), and the degrees of freedom of air pollutants, MT and RH (df = 3, 4, 5) in the DLNM. The model was considered stable if the overall cumulative exposure-response relationship pattern and risk distribution for the risk of hospitalization for genitourinary disorders remained similar over time after varying the df and lag period. All results in this study were considered statistically significantly correlated at a two-tailed p-value of less than 0.05.

## 3. Results

Table 1 shows a summary of the number of daily hospital admissions and meteorological variables for patients with genitourinary disorders in Lanzhou City during the study period. A total of 118,782 patients with genitourinary disorders were hospitalized for treatment in Lanzhou City between January 1, 2014 and December 31, 2020, with an average of 46.5 genitourinary disorders hospitalizations per day, the highest number of hospitalizations per day being 191 and the lowest number being 1, with a large difference. The total number of hospital admissions was 60,485 for male and 58,297 for female, with a male to female ratio of 1.04:1; stratified by age, the total number of hospital admissions was 93268 for <65 years group and 25514 for ≥65 years group. And the mean MT and DTR were 11.1°C (range -12.3 to 30.4°C) and 11.7°C (range 1.0 to 25.0°C). The daily average concentration of PM_10_, SO_2_, and NO_2_ was 112.0 μg/m^3^, 20.6μg/m^3^, 48.2μg/m^3^, respectively.

**Table 1:** Summary of daily genitourinary patient admissions and meteorological variables in Lanzhou, 2014–2020.

Variables	Sum	Mean ± SD	Min	P_5_	P_25_	P_50_	P_75_	P_95_	Max
Total	118782	46.5±32.0	1.0	9.0	17.0	43.0	65.0	111.0	191.0
Male	60485	23.7±17.1	0.0	3.0	9.0	21.0	34.0	57.2	113.0
Female	58297	22.8±15.8	0.0	4.0	9.0	21.0	32.0	55.0	96.0
<65	93268	36.5±25.2	1.0	6.0	14.0	34.0	51.0	87.0	157.0
≥65	25514	10.0±7.7	0.0	1.0	4.0	9.0	14.0	25.0	51.0
PM_10_ (μg/m^3^)	-	112.0±79.8	16.8	39.0	69.6	97.4	133.9	218.7	1484.5
SO_2_ (μg/m^3^)	-	20.6±13.5	3.5	6.0	10.3	16.6	27.2	48.6	80.5
NO_2_ (μg/m^3^)	-	48.2±17.6	10.1	23.8	36.4	46.2	55.7	83.2	151.1
MT (°C)	-	11.1±9.9	-12.3	-4.4	2.3	12.2	19.8	24.9	30.4
DTR (°C)	-	11.7±4.2	1.0	4.6	8.7	11.8	14.6	18.6	25.0
RH (%)	-	51.6±15.1	11.7	27.0	40.1	52.0	62.6	77.0	96.1

**Fig 1:**
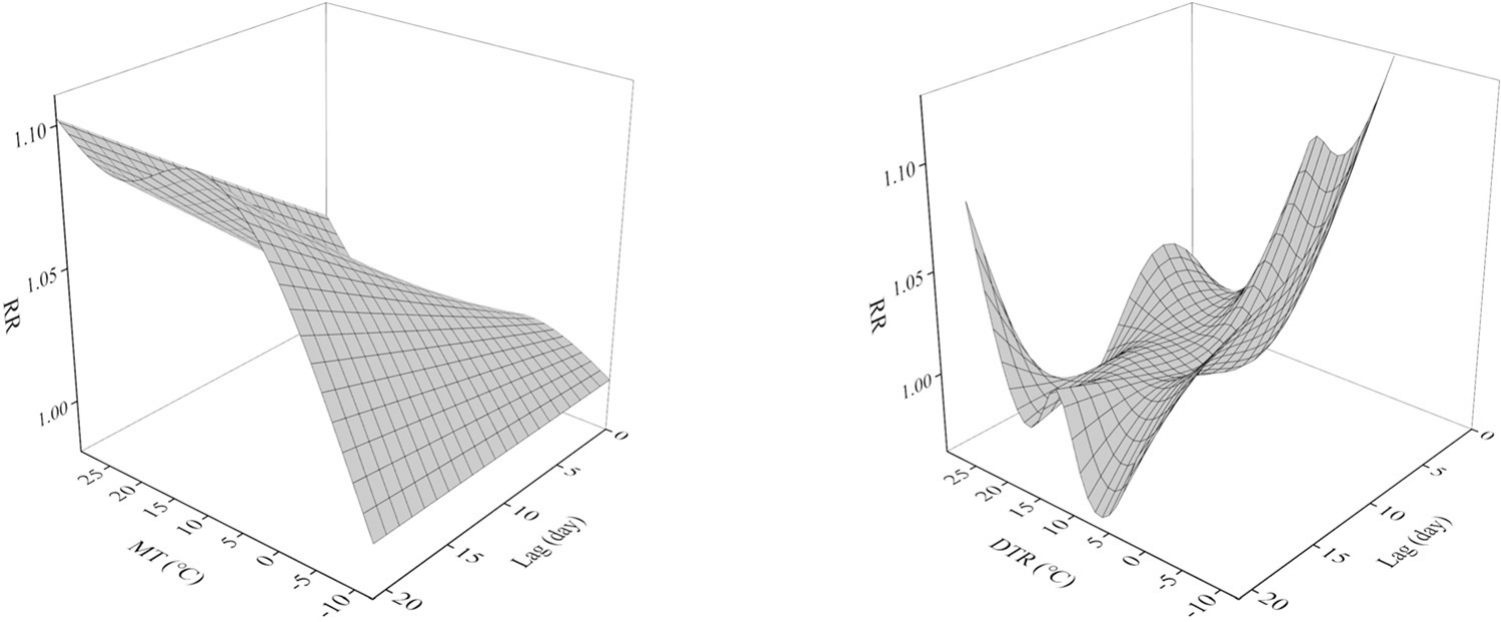
3D plot of MT and DTR relative risk of hospitalization for genitourinary disorders.

**Fig 2:**
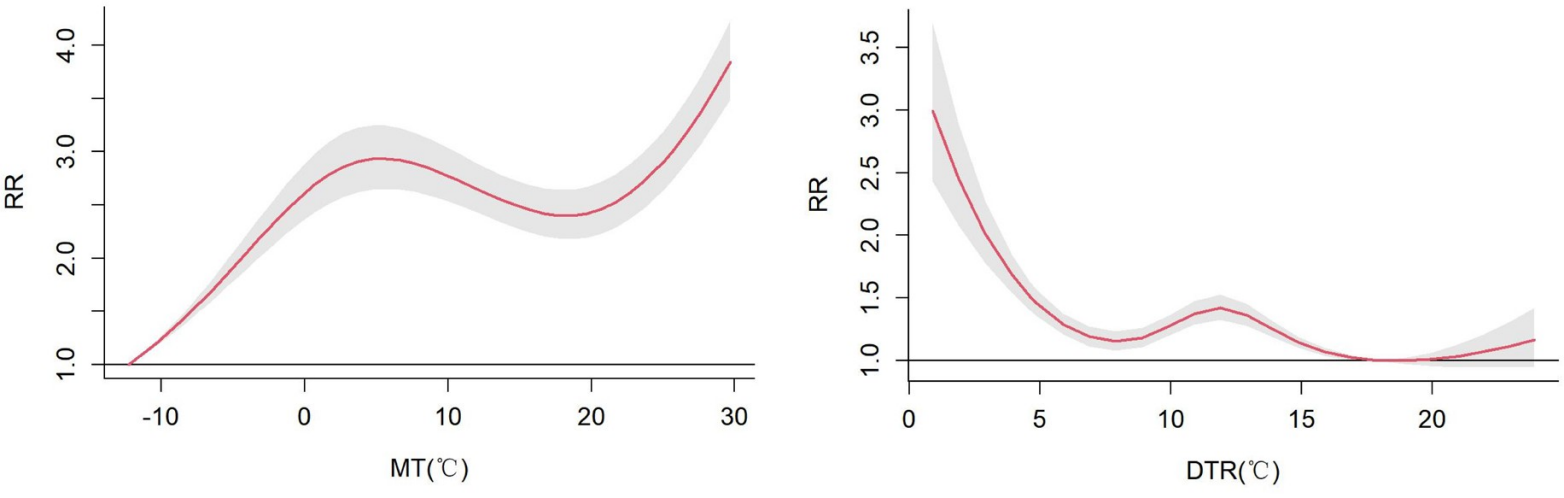
Overall cumulative exposure response curves for MT and DTR in lag0-21. P_5_ of MT: low MT; P_95_ of MT: high MT; P_5_ of DTR: low DTR; P_95_ of DTR: high DTR.

The 3D plot shows the exposure-lag response association between MT and DTR and genitourinary disorders at 0–21 lag days (Fig 1). There is a lagged effect of MT and DTR on the risk of hospitalization for genitourinary disorders and the effect is non-linear. Fig 2 shows the cumulative effect of MT and DTR on the risk of hospitalization for genitourinary disorders at a lag of 0–21 days. The cumulative exposure-response curve for MT on the risk of hospitalization for genitourinary disorders has an “N” shape. MT was deleterious and statistically significant at -11.3~29.7°C. The cumulative exposure-response curve for DTR on the risk of hospitalization for genitourinary disorders has a “W” shape. DTR was harmful and statistically significant at 0.9~16.9°C.

**Fig 3:**
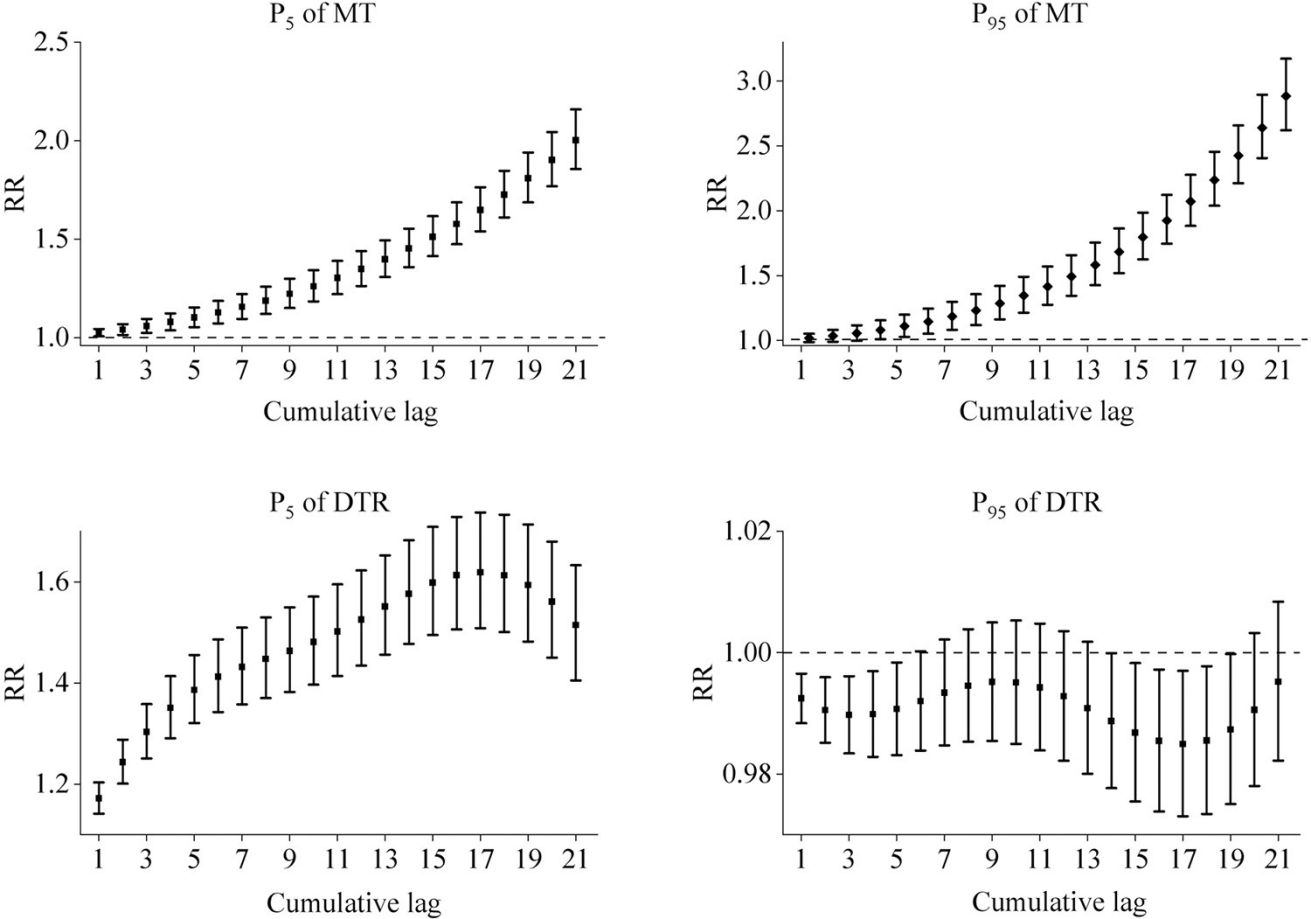
Cumulative lagged response curves for MT and DTR at P_5_ and P_95_ on the risk of hospitalization for genitourinary disorders in the total population. P_5_ of MT: low MT; P_95_ of MT: high MT; P_5_ of DTR: low DTR; P_95_ of DTR: high DTR.

The cumulative lagged effects of MT and DTR at high (P_95_) and low (P_5_) levels on the risk of hospitalization for genitourinary disorders are shown in Fig 3 below. The cumulative lag of low MT showed a dangerous effect at lag0-1 lasting until lag0-21 and the effect was strongest at lag0-21 (RR = 2.001, 95% CI: 1.856~2.159). High MT showed a dangerous effect at lag0-4 lasting until lag0-21 and the effect was strongest at lag0-21 (RR = 2.884, 95% CI: 2.621~3.173). The cumulative lag effect of low DTR showed a hazard effect from lag0-1 to lag0-21, with the strongest effect at lag0-17 (RR = 1.619, 95% CI: 1.508~1.737). None of the cumulative lagged effects of high DTR showed harmful effects.

We also estimated the risk of hospitalization for genitourinary disorders at lag 0–21, and compared it at longer lags by gender and age (S3 Table). It showed that the risk of hospitalization for genitourinary disorders was most affected at lag0-21 days for each 1°C increase in MT. The study found that males were more susceptible to high temperatures than females, with a RR of 1.104 (95% CI: 1.087~1.120), and that those <65 years were more susceptible to high temperatures than those ≥65 years, with an RR of 1.103(95% CI: 1.090~1.116).

**Fig 4:**
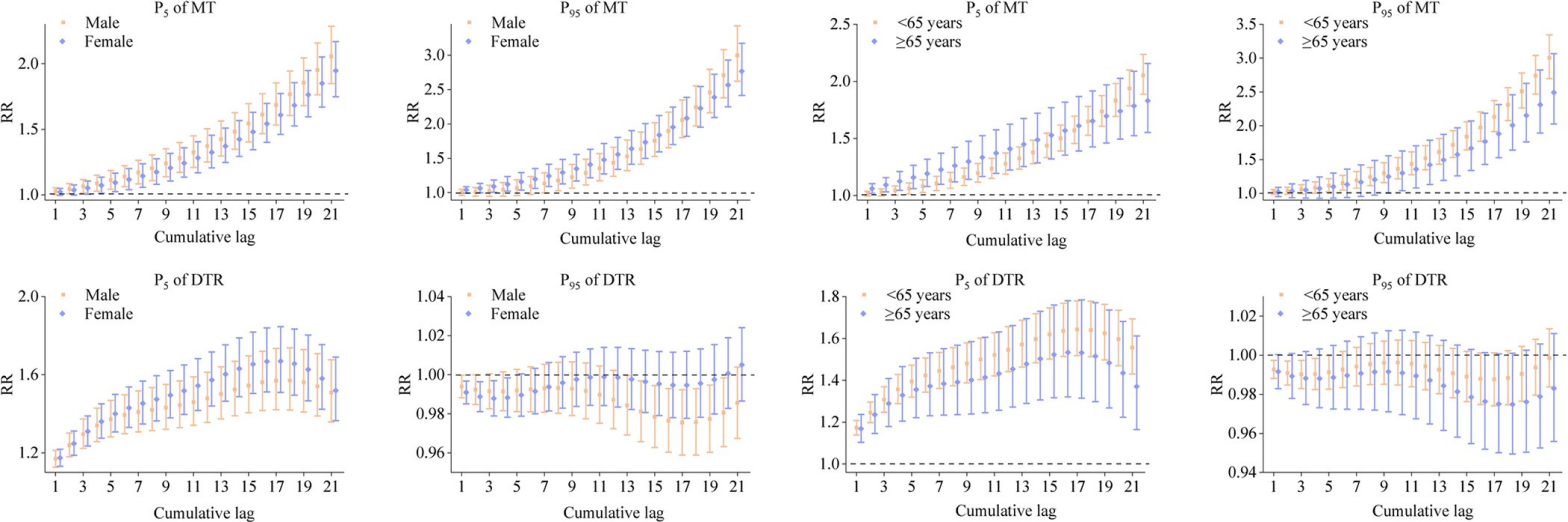
Cumulative lagged response curves for MT and DTR at P_5_ and P_95_ on the risk of hospitalization for genitourinary disorders in different gender and age groups. P_5_ of MT: low MT; P_95_ of MT: high MT; P_5_ of DTR: low DTR; P_95_ of DTR: high DTR.

The cumulative lagged effects of MT and DTR at high (P_95_) and low (P_5_) levels on the risk of hospitalization for genitourinary disorders by gender and age are shown in Fig 4 below. For male, low MT cumulative lag showed the dangerous effect at lag0-1 lasting until lag0-21, and the strongest effect at lag0-21 (RR = 2.055, 95% CI: 1.849~2.285). The high MT cumulative lag showed the dangerous effect at lag0-8 lasting until lag0-21 and the effect was strongest at lag0-21 (RR = 2.998, 95% CI: 2.623~3.427). The cumulative lag effect of low DTR showed the hazard effect from lag0-1 to lag0-21, with the strongest effect at lag0-18 (RR = 1.570, 95% CI: 1.419~1.738). For female, low MT cumulative lag showed risk effect from lag0-3 to lag0-21, and the strongest effect at lag0-21 (RR = 1.946, 95% CI: 1.747~2.168). The high MT cumulative lag showed the dangerous effect at lag0-3 lasting until lag0-21 and the effect was strongest at lag0-21 (RR = 2.767, 95% CI: 2.414~3.172). The cumulative lag effect of low DTR showed a hazard effect from lag0-1 to lag0-21, with the strongest effect at lag0-17 (RR = 1.669, 95% CI: 1.510~1.846).

For those <65 years, the low MT cumulative lag showed the dangerous effect from lag0-3 to lag0-21, and the strongest effect at lag0-21 (RR = 2.054, 95% CI: 1.886~2.237). The high MT cumulative lag showed the dangerous effect at lag0-4 lasting until lag0-21 and the effect was strongest at lag0-21 (RR = 3.003, 95% CI: 2.670~3.344). The cumulative lag effect of low DTR showed the hazard effect from lag0-1 to lag0-21, with the strongest effect at lag0-17 (RR = 1.643, 95% CI: 1.518~1.780). For those aged ≥65 years, low MT cumulative lag showed the dangerous effect from lag0-1 to lag0-21, and the strongest effect at lag0-21 (RR = 1.830, 95% CI: 1.553~2.157). The high MT cumulative lag showed the dangerous effect at lag0-9 lasting until lag0-21 and the effect was strongest at lag0-21 (RR = 2.492, 95% CI: 2.026~3.066). The cumulative lag effect of low DTR showed the hazard effect from lag0-1 to lag0-21, with the strongest effect at lag0-16 (RR = 1.533, 95% CI: 1.321~1.780).

The RR and 95%CI of the risk of hospitalization for genitourinary disorders associated with each 10 μg/m^3^ increase of PM_10_, SO_2_ and NO_2_ concentration under different lag days are presented in S1 Fig. We observed that SO_2_ in single-day lag and NO_2_ in cumulative lag increased the risk of hospitalization for genitourinary disorders.

In the sensitivity analysis, the associations between MT and DTR and the risk of hospitalization for genitourinary disorders remained stable when changing the degrees of freedom of the time variables in the model (df = 6, 7, 8, 9), the maximum lag days (max lag = 21, 14, 28), the degrees of freedom for air pollutants (df = 3, 4, 5), and the degrees of freedom for MT and RH (df = 3, 4, 5) (S2–S4 Figs).

## 4. Discussion

The development of the disease is not only related to the patient’s own qualities, but can also be influenced by changes in temperature. As far as is known from previous surveys, this study is the first to examine the impact of MT and DTR on inpatient treatment of genitourinary disorders in Lanzhou with gender and age subgroup analysis. In the present study, we found that low MT, high MT and low DTR were significantly associated with an increased risk of hospitalization for genitourinary disorders. Although the harmful effects of MT have been demonstrated, the effect of DTR on the risk of hospitalization for genitourinary disorders is relatively new to researchers. This study provides evidence that the effect of DTR is associated with the risk of hospitalization for genitourinary disorders, suggesting that low DTR is an independent risk factor for the risk of hospitalization for genitourinary disorders. The results of this study show some interesting aspects, some of which confirm the results of the literature, and some are compared. Both SO_2_ and NO_2_ had a significant impact on the risk of hospitalization for genitourinary disorders. The Taiwan cohort study found that exposure to higher concentrations of SO_2_ increased the risk of idiopathic nephrotic syndrome [20]. A retrospective study in Hong Kong found that high concentrations of NO_2_ can increase the risk of acute kidney injury admission [21]. The Swedish cohort study found that PM_10_ was not associated with chronic kidney disease at lag0 [22]. The mechanism of SO_2_ and NO_2_ leading to genitourinary disorders, including systemic inflammation, endothelial injury, atherosclerosis, oxidative stress and so on. However, long-term exposure to PM_10_ increases the risk of chronic kidney disease in Taipei [23]. A 6-year outpatient data study in Xi’an found no significant correlation between short-term SO_2_ exposure and outpatient visits for menstrual disorders [24]. These comparison results indicate that not all aspects related to air pollutant factors may be clear. These differences may be due to differences in the geography, climate, etc., in which the study area is located. The results of this study confirm that pollutants increase the risk of hospitalization for diseases of the genitourinary system, so they should be included as con-founders in future analyses of air temperature and diseases.

Previous studies have shown that air temperature-morbidity curves worldwide are almost V-shaped or J-shaped [25, 26]. It also varies according to climatic, geographical and social factors. The cumulative exposure-response curve for MT on the risk of hospitalization for genitourinary disorders was "N" shaped, and high MT was a risk factor for hospitalization for genitourinary disorders, which is consistent with the findings in Chicago, USA, that high temperature has an adverse effect on genitourinary disorders [27]. The N-shaped curve represents the possibility that an increase in the level of exposure to MT may lead to an increase, followed by a decrease, and then an increase in the risk of hospitalization for genitourinary disorders. The shape of this curve suggests a non-monotonic relationship between exposure to MT and the risk of hospitalization for genitourinary disorders. The cumulative exposure-response curve for DTR on the risk of hospitalization for genitourinary disorders is W-shaped, with low DTR being a risk factor for hospitalization for genitourinary disorders. The W-shaped curve indicates that the risk of hospitalization for genitourinary disorders starts to decline with increasing levels of exposure to DTR, followed by a slight upward trend. The shape of this curve suggests that at lower exposure levels, health situation may be poorer and the risk of hospitalization higher.

For some specific respiratory cardiovascular diseases, it is true that there are studies that show an increased risk of seizures and a relatively long lag time in cold temperatures, and a decreased risk of seizures and a relatively short lag time in hot temperatures [2]. However, this pattern contradicts the results of this study, and it may be that this pattern does not apply to all genitourinary disorders or other types of diseases. The length of the lag period depends on the type of disease, individual differences, environmental conditions, and other relevant factors. Each disease has a unique developmental process and lag phase manifestation, and it is not possible to simply establish a universal correlation between lag phase and temperature change.

A global study in 2019 found that both the cold and heat effects were most pronounced in China [28]. Both low MT and high MT were observed to be risk factors for hospitalization for genitourinary disorders when the total population was studied. The results of this study are consistent with other reports. High temperature has been observed as a risk factor for the development of genitourinary disorders in Ganzhou, China [25], Korea [29], Australia [30], USA [31], Italy [32], and Vietnam [33]. The high MT effect is greater than the low MT effect, which is consistent with the finding in Taiwan that the heat effect has a greater effect on genitourinary disorders than the cold effect [26]. The mechanism of the effects of high MT can be understood as the body loses heat through conduction, radiation and evaporation to maintain body temperature equilibrium in a hot environment. This process tends to cause loss of body fluids, the blood becomes relatively concentrated, the viscosity increases, blood pressure is low, small blood vessels throughout the body are in a diastolic state, blood flow is shifted from the internal organs to the skin and muscles, affecting the blood supply to the genitourinary system and thus producing genitourinary disorders. The human body has the ability to adapt to the cold, but within certain limits. When the external ambient temperature is low the decrease in the body’s basal metabolic rate can affect the peripheral and micro-circulation, which in turn can cause organ damage to the genitourinary system. The effect of low MT on disease is thus also a factor that cannot be ignored. However, due to global warming, previous studies have focused more on the effects of high temperatures on disease, and there are fewer studies on the relationship between low MT and genitourinary disorders, with some consistent with the results of this study, such as another study in Korea which also found that low temperatures led to an increased risk of hospitalization for acute kidney injury [5]. Some studies have also concluded that low temperatures are not associated with genitourinary disorders [34], and differences in results may be more related to climatic characteristics and population vulnerability. DTR is the difference between the daily maximum temperature and the daily minimum temperature, representing the intra-day temperature change and predicting the stability of the temperature [35]. In analyses controlling for MT and other confounding factors, low DTR was a risk factor for hospitalization for genitourinary disorders. The effect of high DTR on the risk of hospitalization for genitourinary disorders was not detected in this study. A possible explanation for this cause is people’s adaptation to high DTR [36], as residents of Lanzhou City are exposed to wider DTR for a long period of time, and when there is a drastic change in temperature, people get the relevant weather information through radio, television, and the Internet. When the aforementioned weather phenomena occur, people take certain protective measures, such as increasing clothing and staying indoors, to avoid the effects of high DTR on them. Low DTR is a stable temperature and the mechanism of the effect of DTR on genitourinary disorders is not well elucidated.

In the gender subgroup analysis, different levels of MT and low DTR had an effect on both male and female, suggesting that air temperature affects both genders. High MT was more strongly correlated in male and low DTR was more strongly correlated in female. Possible explanations for female sensitivity to low DTR include, on the one hand, the fact that the female thermoregulatory system is more sensitive to changes in air temperature and is more likely to perceive low DTR. On the other hand, the influence of estrogen and the physiological cycle can make female more sensitive to low DTR [37]. In the age subgroup analysis, those <65 years were more sensitive to low and high MT compared to those ≥65 years, and high MT had a stronger association. This is consistent with previous research findings [9]. The possible reason for this difference may be related to the working age of the <65 years old male population and the higher occupational heat exposure. Moreover, long-term high temperature exposure may lead to fatigue and increased load of the whole body, which makes the resistance of the genitourinary system decrease and is vulnerable to infection and disease. The <65-year-old population was more sensitive to low DTR. This result may be related to the failure of thermoregulation caused by the decrease of temperature variability [38].

## 5. Conclusions

This study provides evidence that the risk of hospitalization for genitourinary diseases is related to air temperature. We found that both low MT and high MT increased the risk of hospitalization for genitourinary disorders in Lanzhou residents, and the effect of high MT was more significant. At the same time, we also found that DTR was independently associated with the risk of hospitalization for genitourinary disorders, especially low DTR. In view of the general trend of global warming, this study has important implications for the prevention and treatment of temperature-related genitourinary diseases, and measures should be taken for different sensitive groups to reduce the impact of air temperature on people’s health.

## Supporting information

S1 Fig RR with 95% CI for the risk of hospitalization genitourinary disorders associated with every 10 μg/m^3^ increase in PM_10_, SO_2_ and NO_2_ concentration, according to single lags ranging from lag 0 to lag 7 days or cumulative lags ranging from lag 0–1 days to lag 0–7 days in single pollutant models. (TIF)

S2 Fig Overall cumulative exposure response curves for MT and DTR at different time variable degrees of freedom. (TIF)

S3 Fig Overall cumulative exposure response curves for MT at different degrees of freedom for meteorological factors, pollutants and maximum lag days. (TIF)

S4 Fig Overall cumulative exposure response curves for DTR at different degrees of freedom for meteorological factors, pollutants and maximum lag days. (TIF)

S1 Table Spearman’s correlation analysis of meteorological factors in Lanzhou City from 2014 to 2020. (DOCX)

S2 Table Q-AIC explained for a variety of combinations of models with different variables and degrees of freedom (df) in the trend and spline terms. (DOCX)

S3 Table The cumulative lag effect of MT on genitourinary disorders, reported as 1°C increase of MT. (DOCX)

## Abbreviations

MT: Mean Temperature
DTR: Diurnal Temperature Range
RH: Relative Humidity
SD: Standard Deviation
DLNM: Distributed Lag Non-linear Models
Q-AIC: Quasi-Akaike Information Criterion
MMT: Minimum Mortality Temperature
df: Degrees of Freedom
RR: Relative Risk
CI: Confidence Interval
