# Supplementary figures and images for: Association between air temperature and risk of hospitalization for genitourinary disorders: An environmental epidemiological study in Lanzhou, China
Source: PLoS One. 2023 Oct 11;18(10):e0292530. doi: 10.1371/journal.pone.0292530 (PMC10566730; doi:10.1371/journal.pone.0292530)

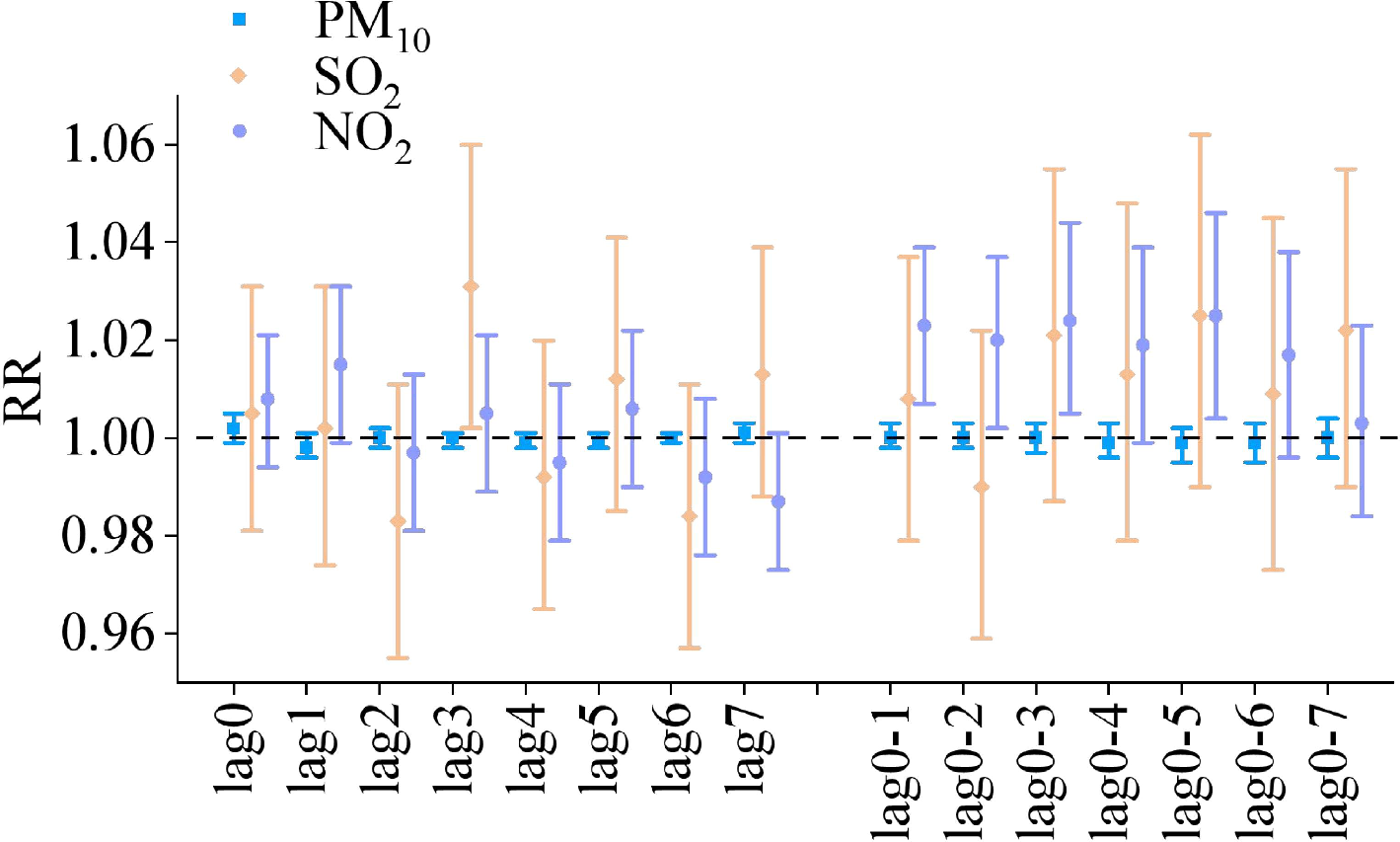

Supplement: S1 Fig — (TIF) [file pone.0292530.s001.tif]

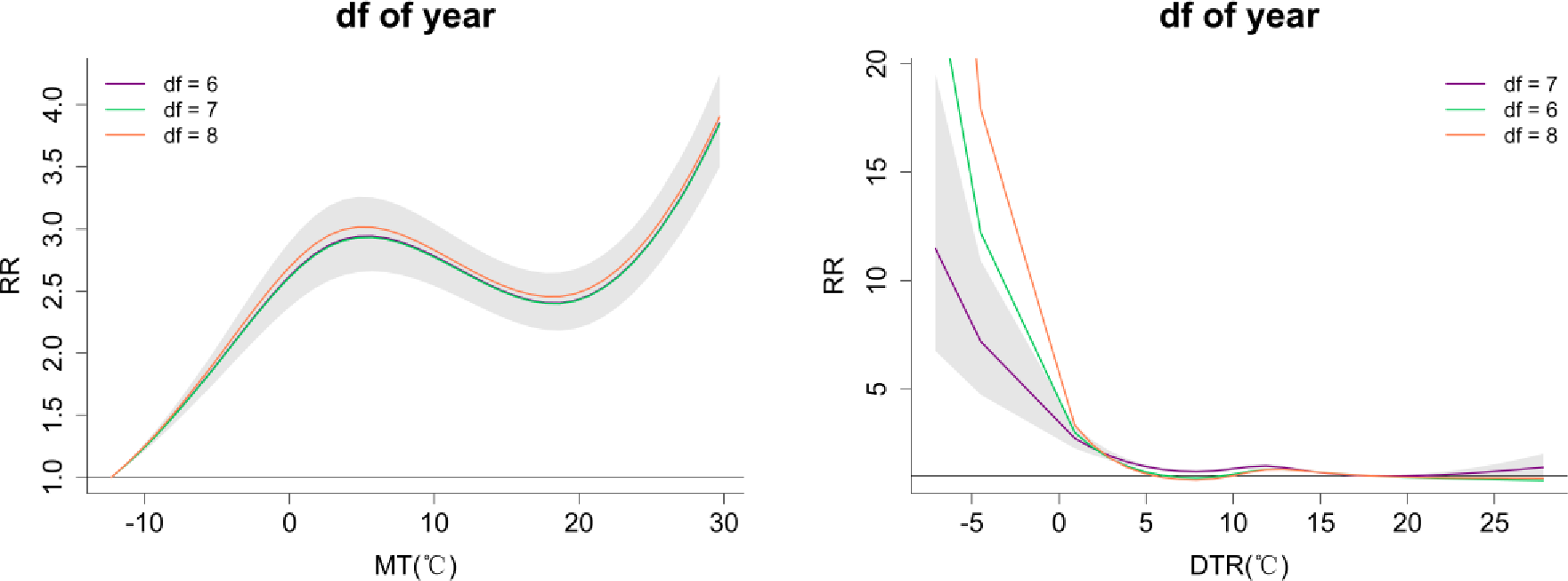

Supplement: S2 Fig — (TIF) [file pone.0292530.s002.tif]

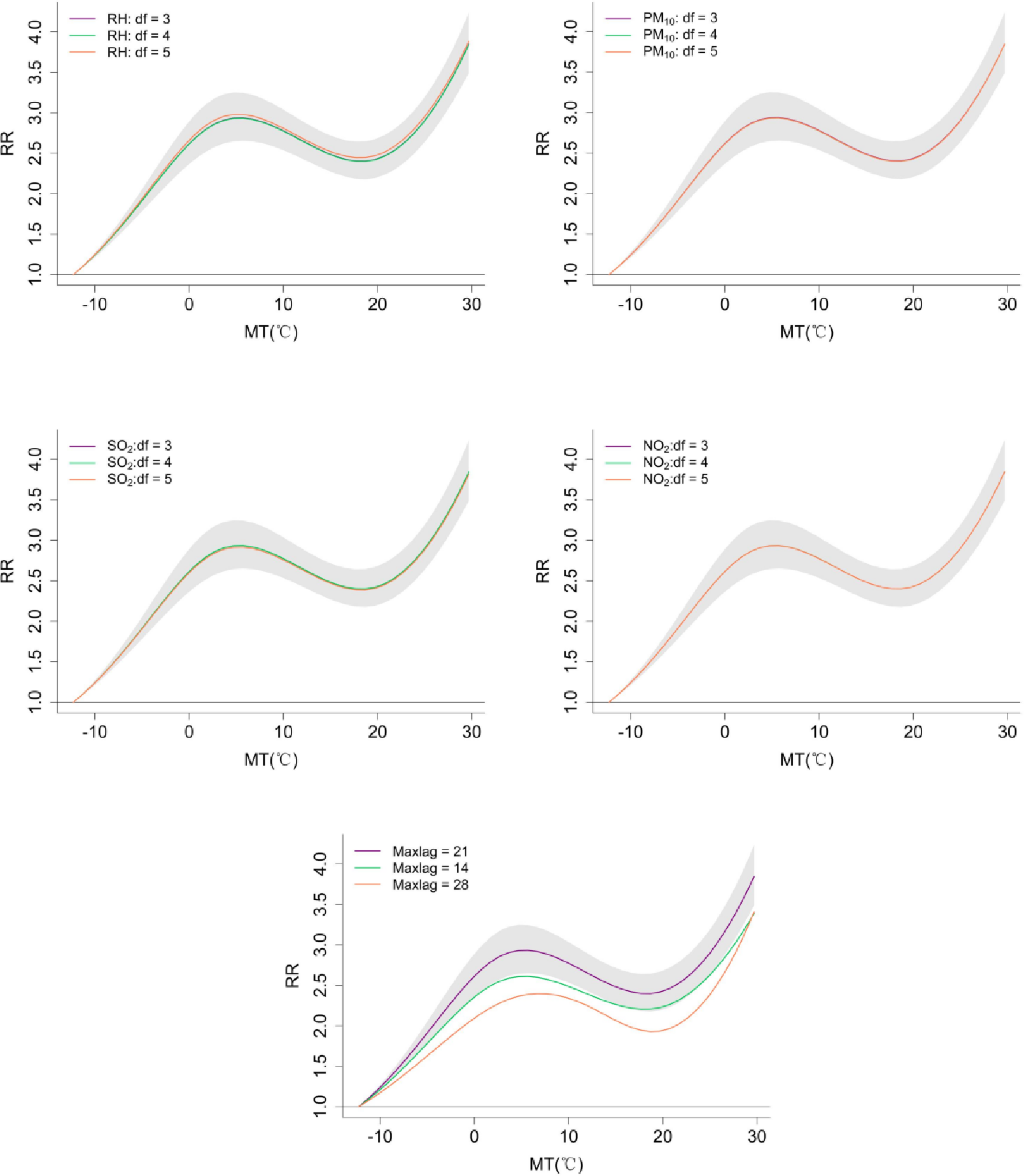

Supplement: S3 Fig — (TIF) [file pone.0292530.s003.tif]

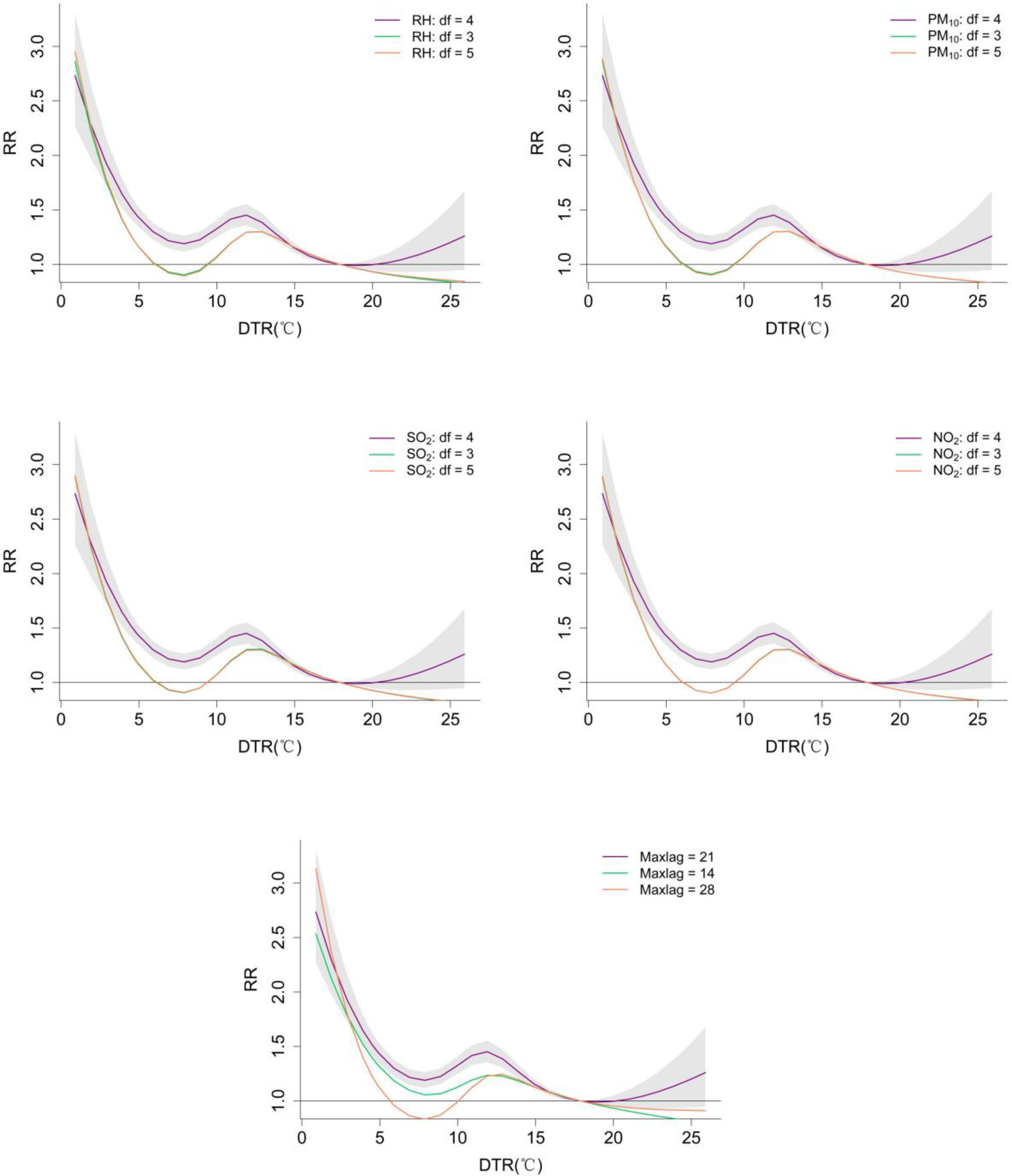

Supplement: S4 Fig — (TIF) [file pone.0292530.s004.tif]
