# Supplementary material for: Association between air temperature and risk of hospitalization for genitourinary disorders: An environmental epidemiological study in Lanzhou, China
Source: PLoS One. 2023 Oct 11;18(10):e0292530. doi: 10.1371/journal.pone.0292530 (PMC10566730; doi:10.1371/journal.pone.0292530)
Supplement: S1 Table — (DOCX) [file pone.0292530.s005.docx]

|  | PM_10_ | SO_2_ | NO_2_ | MT | DTR | RH |
| --- | --- | --- | --- | --- | --- | --- |
| PM_10_ | 1.00 | - | - | - | - | - |
| SO_2_ | 0.65^*^ | 1.00 | - | - | - | - |
| NO_2_ | 0.45^*^ | 0.53^*^ | 1.00 | - | - | - |
| MT | -0.34^*^ | -0.60^*^ | -0.28^*^ | 1.00 | - | - |
| DTR | 0.28^*^ | 0.18^*^ | 0.36^*^ | 0.20^*^ | 1.00 | - |
| RH | -0.42^*^ | -0.26^*^ | -0.17^*^ | -0.03 | -0.41^*^ | 1.00 |

^*^：P<0.05
