# Supplementary material for: Association between air temperature and risk of hospitalization for genitourinary disorders: An environmental epidemiological study in Lanzhou, China
Source: PLoS One. 2023 Oct 11;18(10):e0292530. doi: 10.1371/journal.pone.0292530 (PMC10566730; doi:10.1371/journal.pone.0292530)
Supplement: S2 Table — (DOCX) [file pone.0292530.s006.docx]

| df of lag | df of Time | df of RH | df of air pollution |  | Q-AIC | |
| --- | --- | --- | --- | --- | --- | --- |
|  |  |  |  |  | MT | DTR |
| 3 | 6 | 3 | 3 |  | 31332 | 31116 |
| 3 | 6 | 3 | 4 |  | 31330 | 31111 |
| 3 | 6 | 3 | 5 |  | 31331 | 31114 |
| 3 | 6 | 4 | 3 |  | 31296 | 31092 |
| 3 | 6 | 4 | 4 |  | 31295 | 31088 |
| 3 | 6 | 4 | 5 |  | 31296 | 31090 |
| 3 | 6 | 5 | 3 |  | 31336 | 31121 |
| 3 | 6 | 5 | 4 |  | 31335 | 31115 |
| 3 | 6 | 5 | 5 |  | 31335 | 31118 |
| 3 | 7 | 3 | 3 |  | 31268 | 31082 |
| 3 | 7 | 3 | 4 |  | 31266 | 31076 |
| 3 | 7 | 3 | 5 |  | 31267 | 31080 |
| 3 | 7 | 4 | 3 |  | 31234 | 31058 |
| 3 | 7 | 4 | 4 |  | 31232 | 31053 |
| 3 | 7 | 4 | 5 |  | 31233 | 31055 |
| 3 | 7 | 5 | 3 |  | 31273 | 31087 |
| 3 | 7 | 5 | 4 |  | 31271 | 31081 |
| 3 | 7 | 5 | 5 |  | 31272 | 31084 |
| 3 | 8 | 3 | 3 |  | 31298 | 31100 |
| 3 | 8 | 3 | 4 |  | 31297 | 31095 |
| 3 | 8 | 3 | 5 |  | 31298 | 31098 |
| 3 | 8 | 4 | 3 |  | 31264 | 31076 |
| 3 | 8 | 4 | 4 |  | 31264 | 31072 |
| 3 | 8 | 4 | 5 |  | 31264 | 31074 |
| 3 | 8 | 5 | 3 |  | 31304 | 31105 |
| 3 | 8 | 5 | 4 |  | 31302 | 31099 |
| 3 | 8 | 5 | 5 |  | 31303 | 31102 |
| 4 | 6 | 3 | 3 |  | 31206 | 30912 |
| 4 | 6 | 3 | 4 |  | 31204 | 30904 |
| 4 | 6 | 3 | 5 |  | 31205 | 30909 |
| 4 | 6 | 4 | 3 |  | 31170 | 30888 |
| 4 | 6 | 4 | 4 |  | 31168 | 30881 |
| 4 | 6 | 4 | 5 |  | 31169 | 30885 |
| 4 | 6 | 5 | 3 |  | 31211 | 30916 |
| 4 | 6 | 5 | 4 |  | 31208 | 30908 |
| 4 | 6 | 5 | 5 |  | 31209 | 30912 |
| 4 | 7 | 3 | 3 |  | 31133 | 30863 |
| 4 | 7 | 3 | 4 |  | 31129 | 30854 |
| 4 | 7 | 3 | 5 |  | 31132 | 30859 |
| 4 | 7 | 4 | 3 |  | 31096 | 30839 |
| 4 | 7 | 4 | 4 |  | 31093 | 30830 |
| 4 | 7 | 4 | 5 |  | 31095 | 30834 |
| 4 | 7 | 5 | 3 |  | 31136 | 30867 |
| 4 | 7 | 5 | 4 |  | 31138 | 30863 |
| 4 | 7 | 5 | 5 |  | 31134 | 30878 |
| 4 | 8 | 3 | 3 |  | 31172 | 30879 |
| 4 | 8 | 3 | 4 |  | 31170 | 30871 |
| 4 | 8 | 3 | 5 |  | 31171 | 30875 |
| 4 | 8 | 4 | 3 |  | 31137 | 30856 |
| 4 | 8 | 4 | 4 |  | 31135 | 30848 |
| 4 | 8 | 4 | 5 |  | 31136 | 30852 |
| 4 | 8 | 5 | 3 |  | 31177 | 30883 |
| 4 | 8 | 5 | 4 |  | 31174 | 30874 |
| 4 | 8 | 5 | 5 |  | 31176 | 30879 |
| 5 | 6 | 3 | 3 |  | 31322 | 31040 |
| 5 | 6 | 3 | 4 |  | 31321 | 31034 |
| 5 | 6 | 3 | 5 |  | 31322 | 31037 |
| 5 | 6 | 4 | 3 |  | 31285 | 31015 |
| 5 | 6 | 4 | 4 |  | 31284 | 31011 |
| 5 | 6 | 4 | 5 |  | 31285 | 31012 |
| 5 | 6 | 5 | 3 |  | 31327 | 31044 |
| 5 | 6 | 5 | 4 |  | 31326 | 31038 |
| 5 | 6 | 5 | 5 |  | 31326 | 31041 |
| 5 | 7 | 3 | 3 |  | 31288 | 31008 |
| 5 | 7 | 3 | 4 |  | 31287 | 31002 |
| 5 | 7 | 3 | 5 |  | 31287 | 31005 |
| 5 | 7 | 4 | 3 |  | 31252 | 30984 |
| 5 | 7 | 4 | 4 |  | 31252 | 30979 |
| 5 | 7 | 4 | 5 |  | 31252 | 30981 |
| 5 | 7 | 5 | 3 |  | 31293 | 31012 |
| 5 | 7 | 5 | 4 |  | 31292 | 31006 |
| 5 | 7 | 5 | 5 |  | 31292 | 31009 |
| 5 | 8 | 3 | 3 |  | 31255 | 30998 |
| 5 | 8 | 3 | 4 |  | 31253 | 30992 |
| 5 | 8 | 3 | 5 |  | 31254 | 30994 |
| 5 | 8 | 4 | 3 |  | 31218 | 30973 |
| 5 | 8 | 4 | 4 |  | 31217 | 30968 |
| 5 | 8 | 4 | 5 |  | 31217 | 309970 |
| 5 | 8 | 5 | 3 |  | 31260 | 31002 |
| 5 | 8 | 5 | 4 |  | 31258 | 30996 |
| 5 | 8 | 5 | 5 |  | 31259 | 30999 |
