# Supplementary material for: Association between air temperature and risk of hospitalization for genitourinary disorders: An environmental epidemiological study in Lanzhou, China
Source: PLoS One. 2023 Oct 11;18(10):e0292530. doi: 10.1371/journal.pone.0292530 (PMC10566730; doi:10.1371/journal.pone.0292530)
Supplement: S3 Table — (DOCX) [file pone.0292530.s007.docx]

| Variables | Relative risk (95% CI) | | | | | |
| --- | --- | --- | --- | --- | --- | --- |
|  | Lag0-1 | Lag0-3 | Lag0-5 | Lag0-7 | Lag0-14 | Lag0-21 |
| Total | 1.004(1.001~1.006) | 1.008(1.004~1.013) | 1.014(1.008~1.021) | 1.021(1.013~1.029) | 1.053(1.043~1.063) | 1.099(1.088~1.111) |
| Male | 1.004(1.001~1.008) | 1.009(1.003~1.016) | 1.016(1.007~1.025) | 1.023(1.012~1.034) | 1.056(1.043~1.070) | 1.104(1.087~1.120) |
| Female | 1.003(1.001~1.007) | 1.008(1.001~1.014) | 1.013(1.004~1.022) | 1.019(1.008~1.030) | 1.050(1.036~1.064) | 1.095(1.078~1.111) |
| <65 | 1.003(1.001~1.005) | 1.006(1.001~1.012) | 1.011(1.004~1.019) | 1.018(1.009~1.027) | 1.051(1.040~1.063) | 1.103(1.090~1.116) |
| ≥65 | 1.009(1.003~1.014) | 1.017(1.007~1.027) | 1.025(1.011~1.040) | 1.033(1.016~1.051) | 1.060(1.039~1.082) | 1.085(1.061~1.110) |
